# Common microbehavioral “footprint” of two distinct classes of conditioned aversion

**DOI:** 10.1101/lm.045062.117

**Published:** 2017-05

**Authors:** Emmanouil Paisios, Annabell Rjosk, Evren Pamir, Michael Schleyer

**Affiliations:** Leibniz Institute for Neurobiology (LIN), Department of Genetics of Learning and Memory, 39118 Magdeburg, Germany

## Abstract

Avoiding unfavorable situations is a vital skill and a constant task for any animal. Situations can be unfavorable because they feature something that the animal wants to escape from, or because they do not feature something that it seeks to obtain. We investigate whether the microbehavioral mechanisms by which these two classes of aversion come about are shared or distinct. We find that larval *Drosophila* avoid odors either previously associated with a punishment, or previously associated with the lack of a reward. These two classes of conditioned aversion are found to be strikingly alike at the microbehavioral level. In both cases larvae show more head casts when oriented toward the odor source than when oriented away, and direct fewer of their head casts toward the odor than away when oriented obliquely to it. Thus, conditioned aversion serving two qualitatively different functions—escape from a punishment or search for a reward—is implemented by the modulation of the same microbehavioral features. These features also underlie conditioned approach, albeit with opposite sign. That is, the larvae show conditioned approach toward odors previously associated with a reward, or with the lack of a punishment. In order to accomplish both these classes of conditioned approach the larvae show fewer head casts when oriented toward an odor, and direct more of their head casts toward it when they are headed obliquely. Given that the *Drosophila* larva is a genetically tractable model organism that is well suited to study simple circuits at the single-cell level, these analyses can guide future research into the neuronal circuits underlying conditioned approach and aversion, and the computational principles of conditioned search and escape.

Approaching desirable situations and avoiding undesirable situations are fundamental tasks for any animal, and learning the cues that predict such situations gives an edge in the struggle of life. Animals can learn to avoid situations when these situations feature either something bad or the lack of something good, and to approach situations when they feature either something good or the lack of something bad (for review, see Seymour et al. 2007; Gerber et al. 2014; Kong et al. 2014; see also Discussion). Understanding the processes that bring about conditioned approach or conditioned avoidance requires a model organism that on the one hand provides convenient experimental access for detailed analyses of sensory systems, brain networks, and specific motor actions, and on the other hand is capable of learning-modulated navigation. The larva of the fruit fly *Drosophila melanogaster* arguably is such a model organism. It is a well-established study case in particular for navigation tasks with respect to olfactory cues (Cobb 1999; Louis et al. 2008; Gomez-Marin et al. 2011; Lahiri et al. 2011; Gershow et al. 2012; Gomez-Marin and Louis 2012, 2014; Schulze et al. 2015; Wystrach et al. 2016) as well as for odor-tastant learning (Scherer et al. 2003; Gerber et al. 2009; Diegelmann et al. 2013; Schleyer et al. 2013).

After odor–tastant training, both conditioned aversion and conditioned approach can be observed. Larvae avoid an odor after it has been paired with, for example, the bitter tastant quinine (Gerber and Hendel 2006; Schleyer et al. 2011, 2015a; El-Keredy et al. 2012), or after it has been paired with the lack of, for example, sugar (Gerber and Hendel 2006; Saumweber et al. 2011; Schleyer et al. 2011, 2015a). Likewise, larvae approach an odor after it has been paired with the lack of quinine, or with sugar (Gerber and Hendel 2006; Saumweber et al. 2011; Schleyer et al. 2011, 2015a; El-Keredy et al. 2012). We note that according to Pavlovian terminology, sugar and quinine in such an experiment would be called “appetitive unconditioned stimulus” and “aversive unconditioned stimulus,” respectively. In the *Drosophila* literature, however, “reward” and “punishment” are commonly used instead for many decades (e.g., Tempel et al. 1983). In the following, we will stick to this established terminology of the *Drosophila* community and use “reward” for an appetitive unconditioned stimulus, and “punishment” for an aversive unconditioned stimulus.

Importantly, learned behavior after appetitive and aversive training serves two different functions. Learned behavior after aversive training ceases in the absence of the trained punishment and therefore is best grasped as an escape from something bad, being pointless in the absence of anything to escape from. Learned behavior after appetitive training, in turn, ceases in the presence of the trained reward and therefore may best be viewed as a learned search for the reward that is obsolete in the presence of a sought-for item (for more detailed discussions on this account, see Gerber and Hendel 2006; Schleyer et al. 2011, 2015a). Thus, one can observe two classes of conditioned aversion, namely after paired aversive training and unpaired appetitive training, serving two different functions, namely escape from punishment and search for reward, respectively. Likewise, two classes of conditioned approach can be observed.

Conditioned aversion and approach are commonly measured by counting how the animals distribute themselves in the test arena at the end of a test phase. Only recently, efforts were made to understand the actual modulations of locomotion that underlie conditioned aversion and approach in high resolution (Schleyer et al. 2015b). In this study, we refer to these behavioral modifications as “microbehavior.” While the microbehavioral impact of conditioned approach is relatively well understood (Schleyer et al. 2015b), the microbehavioral impact of conditioned aversion remains unknown. This information gap we seek to close.

We ask whether avoiding in order to escape from something bad and in order to search for something good result from the same modulations of locomotion, and whether these modulations concern the same aspects of locomotion as those found for odor approach (Fig. 1). The results can add to the conceptually interesting comparison of learning about rewards/punishments versus the lack of rewards/punishments. Furthermore, this study can serve as a behavioral framework for the on-going analyses of the underlying circuits (e.g., Berck et al. 2016; Fushiki et al. 2016; Rohwedder et al. 2016). Together, these efforts can provide a detailed picture of how associative memory helps to organize conditioned approach and aversion in a simple nervous system, and how both are implemented in specific motor actions. This study case may then guide future research on how more complex brains organize these processes.

**Figure 1.**
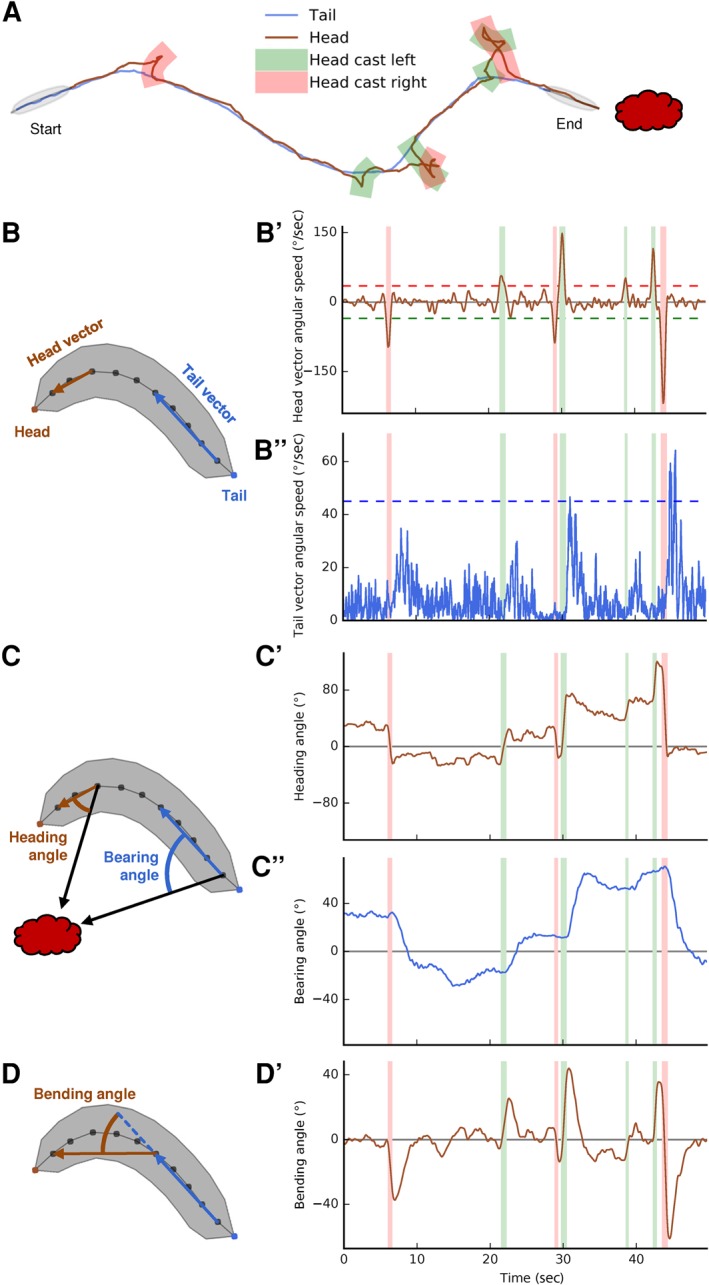
Analysis of larval microbehavior. (*A*) Example of a larva track. Larvae orient through odor gradients by a sequence of relatively straight runs and lateral head movements (head casts, HC). An HC can be either accepted and followed by a change in orientation (e.g., the first two HCs), or rejected and followed by a second HC (e.g., the last few HCs). (*B*–*B*″) HC detection. The larval body is segmented into 12 spine points, from the tail (spine point 1) to the head (spine point 12). Two vectors are defined: the tail vector (spine points 2–6) and the head vector (spine points 9–11). (*B*′–*B*″) The angular speed of the head vector and tail vector, respectively, for the example track displayed in *A*. The stippled lines represent thresholds used to define HCs. Red and green zones correspond to the *left* and *right* HCs in the example track. An HC is detected whenever the angular speed of the head vector exceeds ±35°/sec. Usually, the angular speed of the tail vector increases after an HC because the larval body is following the head's new direction (often called “turn”). (*C*–*C*″) Orientation of the animal. The heading angle measures the orientation of the animal's head relative to the odor source, with positive and negative angles indicating the odor being to the *right* or *left*, respectively. The heading angle changes quickly during HCs and stays relatively stable during runs. The bearing angle measures the orientation of the animal's body relative to the odor source. After each HC or group of HCs, the bearing angle changes with some delay. (*D*–*D*′) Analysis of orientation changes. During an HC, the bending angle increases and reaches a maximum shortly after the end of the HC. When the larva moves straight again, the bending angle drops to zero. Thus, in order to turn *right*, in this example (*A*), a larva first moves its head to the *right* (*B*′), causing a strong bending of the animal (*D*′) and a quick change in the head's orientation toward the negative (*C*′). This is followed by a lateral movement of the back part of the body (*B*″) to align it with the head's new direction (*D*′). This results in a change of orientation and a more negative bearing angle (*C*″).

## Results

**Figure 2.**
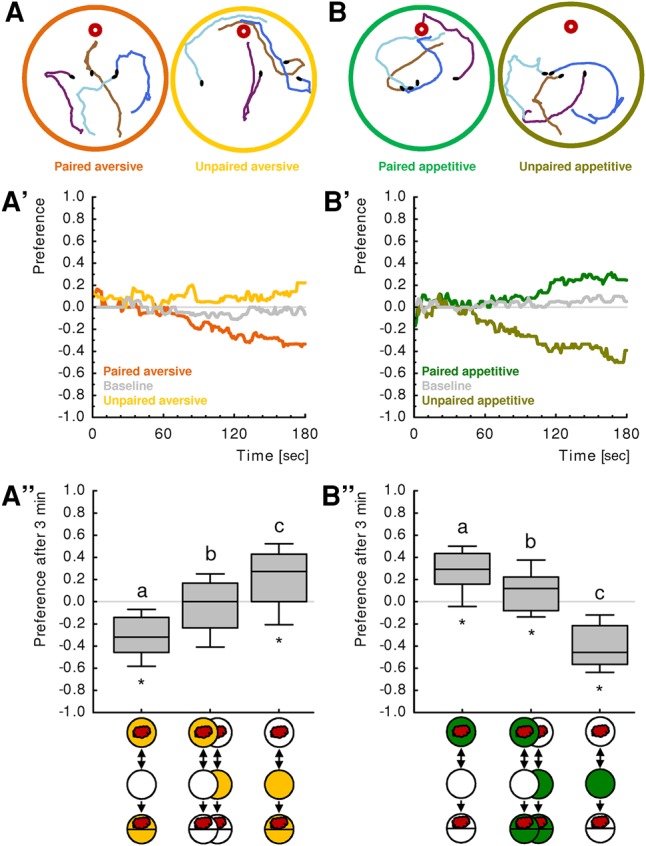
Olfactory preferences after (*A*–*A*″) aversive training and (*B*–*B*″) appetitive training. (*A*,*B*) show example tracks, (*A*′–*B*′) the animals’ preferences over time as detected by the software; (*A*″–*B*″) preferences as based on manual counting after 3 min. (*A*) Representative example tracks of individual animals after paired (*left*) and unpaired (*right*) aversive training. Black dots represent the starting positions of the animals; the small red circle shows the position of the odor source. (*A*′–*A*″) After paired aversive training, larvae avoid the odor whereas after unpaired aversive training (i.e., presenting odor and quinine separately, in consecutive trials) they approach it. When tested in the absence of quinine, learned behavior is not observed; instead, the larvae display an intermediate baseline preference. (*B*) Representative example tracks of individual animals after paired (*left*) and unpaired (*right*) appetitive training. (*B*′–*B*″) After paired appetitive training, larvae approach the odor, whereas after unpaired appetitive training they avoid it. When tested in the presence of fructose, learned behavior is not observed; instead, the larvae display an intermediate baseline preference. Both after appetitive and aversive memory, preference differs across groups (*P* < 0.05, df = 2, *N* = 27, 56, 27, KW). Significant between-group differences (MWU, *P* < 0.05 corrected according to Bonferroni–Holm) are indicated with lower case letters above the boxes, significant differences to chance level (OSS, *P* < 0.05 corrected according to Bonferroni–Holm) are indicated by asterisks below the boxes. For a display of median and 25%–75% quantiles of the time-resolved preferences, see Supplemental Figure S8.

We performed classical conditioning experiments with larval *Drosophila* either using quinine or sugar as punishment or reward, respectively (these are unconditioned stimuli in Pavlovian terminology). First, we determined the group-level preference. We found conditioned odor aversion after paired aversive training (i.e., when odor and quinine punishment were presented simultaneously) (Fig. 2A–A″) as well as after unpaired appetitive training (i.e., when odor and fructose reward were presented separately, in consecutive trials) (Fig. 2B–B″). After unpaired aversive and paired appetitive training, respectively, we found conditioned odor approach (Fig. 2A–A″,B–B″). The difference between paired and unpaired aversive training reflects learned escape from quinine as it was abolished when there was no quinine to escape from; likewise, the difference between paired and unpaired appetitive training reflects learned search for sugar as it was abolished in the presence of the sought-for sugar (Supplemental Fig. S1; Gerber and Hendel 2006; Saumweber et al. 2011; Schleyer et al. 2015a; for a discussion concerning the use of “search” and “escape” in this context, see Schleyer et al. 2011). This offered the opportunity to measure baseline levels of olfactory behavior without the behavioral influence of associative memory (Supplemental Fig. S1; see Schleyer et al. 2015b for further discussion). Given that the measurement of baseline behavior involved testing on different substrates, we asked whether the presence of either the sugar or the quinine substrate also had an influence on innate olfactory preference. We found this not to be the case (Supplemental Fig. S2A), confirming earlier results (Hendel et al. 2005; Schleyer et al. 2011, 2015a,b).

Our results provide in total four experimental groups displaying learned behavior: two groups showing conditioned aversion and two groups showing conditioned approach. Importantly, conditioned aversion can serve two functions: either to search for sugar or to escape from quinine. Hence, by comparing the two groups of animals that show conditioned aversion, we can uncover whether both classes of conditioned aversion are brought about by the same or different microbehavioral features. Likewise, larvae may approach the odor either in search of sugar or to escape from quinine. In the following, we compared the microbehavioral “footprint” (1) of the two classes of conditioned aversion, and (2) of conditioned aversion and conditioned approach. As larvae orient through odor gradients by a sequence of relatively straight runs and lateral head movements (head casts, HC), which are followed by changes in orientation (Fig. 1A), we focused on three features of chemotaxis: how fast larvae run, under which circumstances they initiate a HC, and where-to they direct the HC.

**Figure 3.**
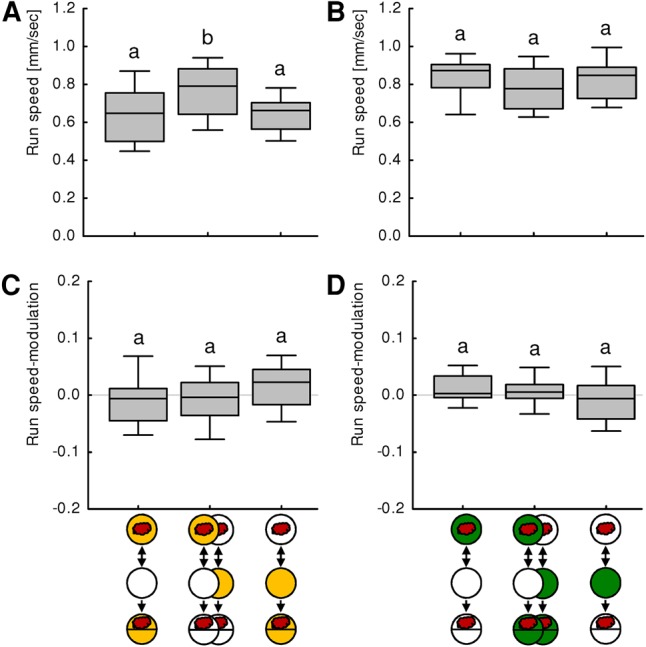
Analysis of run speed. (*A,B*) show run speed, (*C,D*) show run speed-modulation. (*A*) After aversive training, both reciprocally trained groups tested in the presence of quinine display lower run speed than the baseline, but do not differ from each other. (*B*) After appetitive training, the reciprocally trained groups tested on pure agarose display the same run speed, which is tendencially higher than under baseline conditions, i.e., in the presence of fructose. (*C*) After aversive training, there is no significant effect of training experience on run speed-modulation, and the pooled values of run speed-modulation are not different from zero (OSS, *P* > 0.05). That is, larvae do not modulate their run speed depending on their orientation to the odor. (*D*) After aversive training, there is no significant effect of training experience on run speed-modulation, and the pooled values of run speed-modulation are not different from zero (OSS, *P* > 0.05). Only with respect to run speed after aversive training did we find a significant difference across groups (*P* < 0.05, df = 2, *N* = 27, 56, 27, KW), which was, however, not associative in kind as both the reciprocally trained groups were affected. In all other cases, no significant difference across groups was detected (*P* > 0.05, df = 2, *N* = 27, 56, 27, KW). Significant between-group differences (MWU, *P* < 0.05 corrected according to Bonferroni–Holm) are indicated by lower case letters above the boxes.

We found no effect of associative training experience on run speed (Fig. 3A,B). Thus, neither conditioned aversion nor conditioned approach comes about by general increases or decreases of run speed. On the contrary, run speed was decreased in the presence of quinine (left and right box in Fig. 3A). Also in presence of sugar run speed is slightly yet statistically nonsignificantly reduced (middle box in 3B). A reduction in presence of either substrate was also found in experimentally naïve larvae (Supplemental Fig. S2B). With respect to sugar, this observation could be a hint that gustatory behavior may be best viewed as a form of kinesis (Fraenkel and Gunn 1961): in a gustatory “good” situation, slowing down helps the larva not to drift away from a food source. However, following this logic one would expect that larvae would speed up in the presence of quinine in order to escape—but in fact they slow down. The observed effects on speed may rather be based on changes in the physical properties of the substrate caused by adding sugar or quinine (stiffness, structure of the surface, etc.; notably, agarose concentration has also been found to affect run speed [Apostolopoulou et al. 2014]), or may reflect a strategy of the larvae for discerning tastant gradients.

Next, we tested whether larvae may modulate their run speed depending on their orientation to the odor source, and whether such modulation may differ due to training experience. Although after paired aversive and unpaired appetitive training, larvae tended to run faster while heading away from the odor than while heading toward it, whereas after unpaired aversive and paired appetitive training the opposite trend was observed (Fig. 3C,D), these trends were small and not significant. We conclude that modulations of run speed are no major determinants of conditioned aversion or conditioned approach.

**Figure 4.**
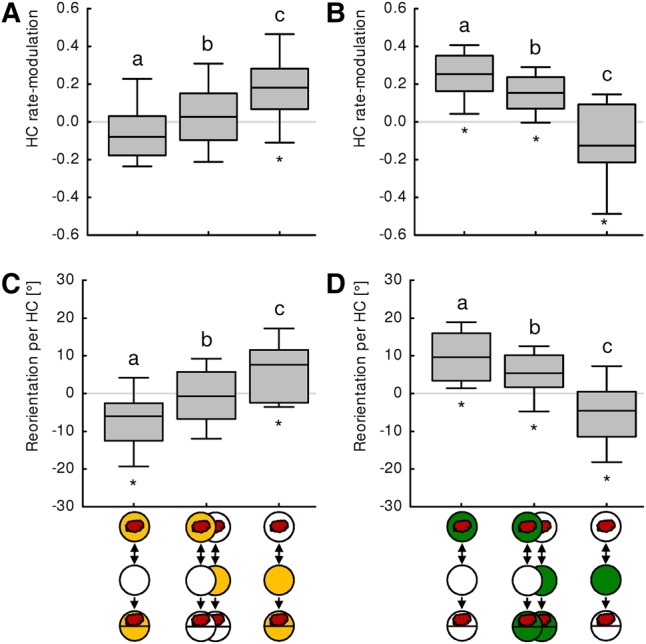
Analysis of head-casting. (*A*,*B*) show the HC rate-modulation and (*C*,*D*) the Reorientation per HC. (*A*) After paired aversive training, larvae tend to perform more HCs when heading toward the odor than away from it, yielding a slightly negative HC rate-modulation. After unpaired aversive training, in contrast, more HCs are performed when larvae head away from the odor, resulting in a positive HC rate-modulation. When tested in the absence of quinine, the larvae display an intermediate baseline HC rate-modulation. (*B*) HC rate-modulation is positive after paired appetitive training, and slightly negative after unpaired appetitive training. When tested in the presence of fructose, the larvae display an intermediate baseline HC rate-modulation. (*C*) After paired aversive training, larvae after an HC reorient themselves more away from the odor than toward it, indicated by negative reorientation values. After unpaired aversive training, in turn, larvae reorient themselves more toward the odor. When tested in the absence of quinine, the larvae display an intermediate baseline Reorientation per HC. (*D*) After paired appetitive training, larvae after an HC reorient themselves more toward the odor, whereas after unpaired appetitive training, they reorient themselves more away from the odor. When tested in the presence of fructose, the larvae display an intermediate baseline Reorientation per HC. All displayed features of chemotaxis differ across groups (*P* < 0.05, df = 2, *N* = 27, 56, 27, KW). Significant between-group differences (MWU, *P* < 0.05 corrected according to Bonferroni–Holm) are indicated with lower case letters above the boxes, significant differences to chance level (OSS, *P* < 0.05 corrected according to Bonferroni–Holm) are indicated by asterisks below the boxes.

In contrast to run speed, HC initiation was strongly modulated affected by associative experience. In both experimental groups displaying conditioned aversion (after paired aversive and unpaired appetitive training), the larvae tend to perform more HCs when heading toward the odor than when heading away from it, a behavior that upon the used convention shows as negative HC rate-modulation (Fig. 4A,B). Conversely, in both experimental groups displaying odor approach (after unpaired aversive and paired appetitive training), the larvae performed fewer HCs when heading toward the odor than when heading away, yielding a positive HC rate-modulation (Fig. 4A,B).

A similar picture emerges regarding the direction of the HCs. In both groups displaying conditioned aversion (after paired aversive and unpaired appetitive training), the larvae reoriented themselves after a HC more likely away from the odor than toward (Fig. 4C,D). In both groups displaying odor approach (after unpaired aversive and paired appetitive training), in contrast, the larvae reoriented themselves after a HC more likely toward the odor than away from it (Fig. 4C,D).

Thus, avoiding an odor in order to escape from quinine and in order to search for sugar affected HC initiation and HC direction in the same way. These modulations were of opposite sign compared with the effects of conditioned approach.

## Discussion

In this study, we investigated how two classes of odor aversion are microbehaviorally implemented in specific motor actions. In our experiments, larvae avoided an odor either because it predicted quinine that the larvae sought to escape from, or because it predicted the lack of sugar that the larvae were searching for (Fig. 2). Also in adult flies, vertebrates and humans, conditioned aversion and conditioned approach can have different causes: a cue can be avoided because it predicts a punishment, or the end, or a lack of a reward (sometimes referred to as “frustration learning”), and it can be approached because it predicts a reward, or the end of a punishment (called “relief learning”), or the lack of a punishment (called “safety learning”) (Rescorla and Wagner 1972; Dickinson and Dearing 1979; Gray 1987; Tanimoto et al. 2004; Rogan et al. 2005; Kim et al. 2006; Andreatta et al. 2010; Leknes et al. 2011; Navratilova et al. 2013; Marshall et al. 2014; Mohammadi et al. 2014; for review, see Seymour et al. 2007; Gerber et al. 2014; Kong et al. 2014). Although these different types of memories can be discriminated conceptually, the question arises to which extent the lack of a reward can be seen as just another kind of punishment, and likewise the lack of punishment as just another kind of reward. Our results show that the two classes of conditioned aversion observed in our experiments are strikingly alike—at the level of specific motor actions. In both cases larvae tend to perform more HCs when heading toward the odor than when heading away (Fig. 4A, left; Fig. 4B, right) and direct their HCs away from the odor rather than toward it (Fig. 4C, left; Fig. 4D, right). Likewise, the two classes of odor approach we observed share the same effects on chemotaxis, and are of opposite sign as compared with aversion (Fig. 4). From this perspective, the two classes of aversion are very similar.

On the other hand, as discussed earlier, the two classes of conditioned aversion serve different functions: either to escape from quinine or to search for sugar (Gerber and Hendel 2006; Schleyer et al. 2011). Importantly, they can be discriminated experimentally: larvae will avoid a quinine-predicting odor only in the presence of quinine (Fig. 2A). In contrast, larvae will avoid an odor that predicts the lack of sugar only in the absence of sugar (Fig. 2B). Thus, on a tasteless substrate that contains neither quinine nor sugar, larvae avoid an odor that predicts the lack of sugar, but they do not avoid an odor that predicts quinine (compare Fig. 2A″, middle box, and Fig. 2B″, right box; see also Schleyer et al. 2011). From this perspective, the two classes of aversion are clearly distinct.

To which extent are the neuronal substrates of aversive and appetitive memories in *Drosophila* shared or distinct? The current working hypothesis for associative olfactory learning in fruit flies and larvae locates the memory trace in the output synapses of the mushroom body Kenyon cells (for review, see Heisenberg 2003; Gerber et al. 2009; Diegelmann et al. 2013; Schleyer et al. 2013). The Kenyon cells receive on the one hand odor information, on the other hand reward and punishment signals. Thus, to this extent aversive and appetitive memories in *Drosophila* share the same circuit. Rewards (appetitive unconditioned stimuli in Pavlovian terminology) are signaled by a subset of dopaminergic neurons (Rohwedder et al. 2016), similar to the case in vertebrates (Schultz 2015). Whether dopaminergic neurons in *Drosophila* convey a prediction error signal rather than the reward signal per se as implicated by learning theory (Rescorla and Wagner 1972) and demonstrated in vertebrates (Schultz 2015) remains to be shown in *Drosophila*.

**Figure 5.**
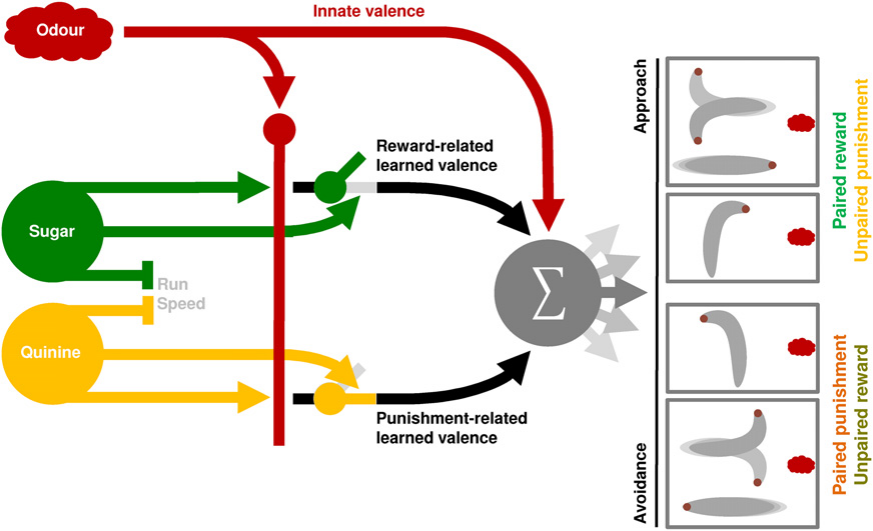
Working hypothesis for how conditioned odor aversion and approach come about. Both sweet and bitter substrates reduce run speed. Odor valence is formed by the sum of the odor's innate valence (which is usually positive in the case of larval *Drosophila*) and learned valence resulting from associative memory (see also Wystrach et al. 2016). Learned valence can be negative after paired aversive or unpaired appetitive training, and positive after unpaired aversive or paired appetitive training. Notably, signaling of learned valence after appetitive training (be it positive or negative) is blocked by the presence of the reward. Learned valence after aversive training, in contrast, is signaled only in the presence of the punishment. Signaling of innate valence is not affected by the presence of reward or punishment (see also Supplemental Fig S2A). After paired aversive or unpaired appetitive training, a negative learned valence is added to the innate valence of the odor, which may in sum lead to conditioned aversion. Consequently, more HCs are performed while heading toward the odor than while heading away, and HCs are directed away from rather than toward the odor. Notably, if the positive innate valence of an odor happens to be higher than the learned negative valence, larvae may not show net aversion (Schleyer et al. 2015b). After unpaired aversive or paired appetitive training, the summed innate and learned valences may lead to approach through a bias toward performing more HCs while heading away from the odor than while heading toward it. Moreover, HCs are directed toward rather than away from the odor.

Punishments (aversive unconditioned stimuli in Pavlovian terminology) are signaled by another subset of dopaminergic neurons in the fruit fly (Schroll et al. 2006; Liu et al. 2012; Aso et al. 2014a,b; Rohwedder et al. 2016), which is controversial in vertebrates (Lammel et al. 2012; Schultz 2013). Thus, different sets of dopaminergic neurons were found to be necessary in *Drosophila* appetitive or aversive learning, respectively (e.g., Schroll et al. 2006; Liu et al. 2012; Aso et al. 2014a,b; Rohwedder et al. 2016). These different sets of dopaminergic neurons innervate clearly separated compartments of the mushroom body (Pauls et al. 2010; Aso et al. 2014a). From the mushroom body, appetitive and aversive memory information is signaled by different mushroom body output neurons, gathering input from different mushroom body compartments, toward motor control (Aso et al. 2014b). Thus, appetitive and aversive memories rely on different neuronal pathways on the level of dopaminergic neurons and mushroom body output neurons, and are likely to be summed up only downstream from the mushroom body output neurons (Fig. 5). Whether the same or different dopaminergic neurons, and the same or different mushroom body output neurons are involved in paired and unpaired aversive training (which could be interpreted as punishment and safety learning, respectively), remains unknown (the same applies to paired and unpaired appetitive training).

The output neurons of the mushroom body thus are thought to code the learned valence of an odor. This learned valence then can be added to the odor's innate valence and accordingly shift behavior toward more approach or more aversion—depending on the kind of odor and the kind of memory associated with it (Fig. 5; Aso et al. 2014b; Owald et al. 2015; Schleyer et al. 2015b). After paired aversive and unpaired appetitive training, the learned valence is negative, shifting the behavior toward aversion. After paired appetitive and unpaired aversive training, in turn, the learned valence is positive, shifting the behavior toward approach.

Which individual mushroom body output neurons signal which type of learned valence, and how the integration of innate and learned valence is organized downstream from the mushroom body output neurons is the subject of on-going research. Such circuit-level understanding will give us insights into how a relatively simple nervous system tackles the tasks of approach and aversion, and hopefully also help us to understand how these processes are organized in more complex systems. Given that autonomous agents and robots also face the same crucial tasks of avoiding unfavorable situations and approaching desirable ones, the *Drosophila* larva could act as a model for tackling these issues.

## Materials and Methods

### Animals, reinforcement, and odors

Feeding-stage, third instar *Drosophila melanogaster* larvae five days after egg laying from Canton-S wild-type were used. The larvae were maintained on standard fly food at 25°C, 60% relative humidity, in a 12 h light–dark cycle. The experiments used Petri dishes of an inner diameter of 9 and 15 cm for training and test, respectively, which were prefilled with 1% agarose (electrophoresis grade; Roth) and stored at 4°C until used. 2 mol/L fructose (FRU; CAS: 57-48-7; purity 99%; Roth) or 5 mmol/L quinine hemisulfate (QUI; CAS: 6119-70-6; Sigma-Aldrich) were added to the agarose as reward and punishment, respectively. Please note that the terms “reward” and “punishment” are frequently used in *Drosophila* learning and memory literature instead of the Pavlovian terminology of “appetitive unconditioned stimulus” and “aversive unconditioned stimulus,” respectively.

As odor, *n*-amyl acetate (AM; CAS: 628-63-7; Merck) was diluted 1:20 in paraffin oil (CAS: 8012-95-1; Sigma-Aldrich) and presented in custom-made Teflon containers (5 mm diameter), covered by perforated lids.

### Learning experiments

Associative learning experiments followed established protocols (Gerber and Hendel 2006; Saumweber et al. 2011; Schleyer et al. 2015a,b). For aversive training, one group of larvae was trained such that *n*-amyl acetate (AM) was presented simultaneously with the quinine substrate for 2.5 min, immediately followed by a blank trial on a Petri dish with empty Teflon containers (EM) and without quinine for another 2.5 min (paired training). In a second experimental group, the larvae were trained such that AM and quinine were presented on separate Petri dishes (unpaired training). This training cycle was performed three times. Across repetitions of the experiment, in half of the cases the odor-containing trial came first (AM + Quinine/EM and AM/EM + Quinine, respectively), in the other half of the cases the sequence was reversed (EM/AM + Quinine and EM + Quinine/AM, respectively). For the subsequent test, the animals were placed in the middle of a 15-cm-diameter Petri dish with an AM-loaded container on one side and an empty container on the other side in order to create a choice situation. Larval behavior was recorded using a camera and analyzed offline as described below.

For appetitive learning experiments, fructose was used instead of quinine. Analogous to the aversive protocol described above, odor and fructose were presented either paired or unpaired, and testing and recording were performed as mentioned.

The test Petri dishes may or may not contain a taste reinforcer as mentioned in the results. For all experiments, experimenters were blind with respect to the test conditions.

### Data analysis

After the 3 min of test, we determined the number of animals on the odor side (#AM), the number on the no-odor side (#EM), and the number of larvae on a 1-cm-wide middle stripe (#middle). From this, we calculated the odor preference [−1; 1] as:

(1) $$\text{Preference}=\frac{\#\text{AM}-\#\text{EM}}{\#\text{Total}}.$$

During the test, we recorded larval behavior using a camera (Basler acA2040-90um). These videos were analyzed using custom-written analysis software based on the approach described in Schleyer et al. (2015b). The most salient change relative to that study was the use of head casts (HC) instead of turns to characterize chemotaxis. Larvae orient in odor gradients by a sequence of rather straight runs, HCs and turns (Fig. 1A). HCs are used to scan for local differences in odor concentration by laterally moving the head. An HC can either be rejected, resulting in another HC (usually in the opposite direction), or accepted, resulting in the body starting to run straight again. The body hence follows the new direction of the head; such an accepted HC is observed as a turn (Fig. 1). As such a turn is by definition preceded by an HC and the majority of HCs are in the same direction as the subsequent turn (Gomez-Marin et al. 2011), we decided to use exclusively HCs for the present analysis.

An HC was detected whenever the angular speed of the animal's head vector (Fig. 1B′) exceeded a threshold of 35°/sec and ended as soon as it dropped below that threshold again. If the angular speed of the tail vector (Fig. 1B″) at the same time exceeded a threshold of 45°/sec, this event was not regarded as an HC, but rather taken to indicate a rotation of the larval body. In accordance with previous work (Schleyer et al. 2015b), we took into account only HCs with an HC angle >20° (for a definition of the HC angle see below, for data on HCs with an HC angle <20° see Supplemental Fig. S3). The time when an animal was not head-casting was regarded as a run, discarding 1.5 sec before and after an HC to exclude decelerating and accelerating phases that usually happen before and after an HC, respectively.

We calculated the following variables:

- Bearing angle toward odor: the orientation an animal's body relative to the odor source. 0° indicates that the odor is in front of the larva; positive and negative angles indicate that the odor is to the right or left, respectively; ±180° indicates that the odor is to the rear (Fig. 1C).
- Heading angle toward odor: as bearing angle, but measuring the orientation of the animal's head relative to the odor source (Fig. 1C).
- Run speed: the average speed (mm/sec) of the larval midpoint during runs. To quantify a potential modulatory effect of the bearing on run speed, we introduced “run speed-modulation” as the run speed while heading toward the odor minus the run speed while heading away from the odor, divided by the sum of both:
  (2) $$\text{Run speed-modulation}=\frac{\text{Run speed toward}-\text{Run speed away}}{\text{Run speed toward}+\text{Run speed away}}.$$
  Thus, if animals would modify their run speed such that they would speed up whenever they head away from the odor (absolute bearing angle >90°) and slow down whenever they head toward an odor (absolute bearing angle <90°) we would obtain a negative Run speed-modulation.
- HC initiation: we measured a HC rate as the number of HCs (#HC) divided by the duration of time the larvae were tracked (T):
  (3) $$\text{HC rate}(\text{HC/s})=\frac{\text{sum}(\#\text{HC})}{\text{sum}(\text{T})}.$$
  For a display of the HC rate, see Supplemental Figure S4A,B. Animals that showed odor aversion had a high HC rate when they were heading toward the odor (absolute bearing angle <90°) and a lower HC rate when they were heading away from the odor (absolute bearing angle >90°). To quantify this modulatory effect of the bearing, we introduced “HC rate-modulation” as the HC rate while heading away from the odor source minus the HC rate while heading toward the source, divided by the sum of both:
  (4) $$\text{HC rate-modulation}=\frac{\text{HC rate away}-\text{HC rate toward}}{\text{HC rate away}+\text{HC rate toward}}$$
  Thus, in the described example case of odor aversion (HC rate away < HC rate toward) we would obtain a negative HC rate-modulation.
- HC direction: for each time point we determined an animal's bending angle (BA) as the angle between the tail vector and a vector from spine points 6–11 (Fig. 1D). Then, an HC angle was calculated as the difference in the bending angle before and after an HC:
  (5) HC angle(°)=BA after HC−BA before HC.
  For a display of the HC angle, see Supplemental Figure S4C,D.

In order to statistically compare the direction of HCs with respect to the odor source across groups, we determined the Reorientation per HC with respect to the odor. That is, we subtracted the angular deviation of the animal's head vector from the odor (as measured by the absolute value of the heading angle, HA, see Fig. 1C) after an HC from its angular deviation before the HC:

(6) $$\begin{array}{ccc} \text{Reorientation per HC} & = & \text{abs}(\text{HA before HC})\\ & & -\text{abs}(\text{HA after HC}). \end{array}$$

If an HC was directed away from the odor source, the heading angle after the HC would be higher than before, thus resulting in a negative reorientation value. The use of the heading angle instead of the bearing angle is warranted because the bearing angle after an HC changes only with some delay, see Fig. 1C″.

All these calculations were performed once per Petri dish. That is, in the data presented as box plots the sample size *N* equals the number of test Petri dishes, each containing approximately 20 animals.

To visualize how the different aspects of locomotion vary with both distance to the odor source and bearing toward odor we pooled the data from all Petri dishes for each experimental condition, applied a sliding box filter of ±60° and ±23 mm at each step (step width of 6° and 2.3 mm), and plotted the aggregated values of run speed (Supplemental Fig. S5), HC rate (Supplemental Fig. S6), and HC angle (Supplemental Fig. S7). It turned out that larval behavior differed with respect to the distance from the odor and that in particular aversion is mainly observed when the animals are relatively close to the odor (Supplemental Figs. S6, S7). Therefore we restricted all analyses (except Supplemental Figs. S5–S7) to animals near the odor source (distance <59 mm, which is half the maximal distance from the odor source).

### Statistics and graphs

Nonparametric statistics (one-sample sign test, Kruskal–Wallis test, Mann–Whitney *U*-test; OSS, KW, MWU) were applied throughout the study, using Statistica (StatSoft) for the PC (for one-sample sign-tests we use a custom-written function for Excel [Microsoft]). When multiple comparisons were performed within one analysis, a Bonferroni–Holm correction was applied to keep the experiment-wide error rate below 5% (Holm 1979). When data are displayed as box plots, the middle line shows the median, the box boundaries the 25% and 75% quantiles, and the whiskers the 10% and 90% quantiles.

### Ethics

Procedures comply with applicable law.

### Data accessibility

Data sets were uploaded on a repository and will be made publicly available upon publication.

## Competing interest statement

The authors declare no competing interests.

## Supplementary Material

Supplemental Material
