# Supplementary material for: Common microbehavioral “footprint” of two distinct classes of conditioned aversion
Source: Learn Mem. 2017 May;24(5):191–8. doi: 10.1101/lm.045062.117 (PMC5397685; doi:10.1101/lm.045062.117)
Supplement: Supplemental Material [file supp_24_5_191__index.html]

Supplemental Material 

# Common microbehavioral “footprint” of two distinct classes of conditioned aversion

## Supplemental Material

- Supplemental\_Figure\_Legends.docx
- Supplemental\_FigureS7.tiff
- Supplemental\_FigureS8.tiff
- Supplemental\_Movie\_1.mp4
- Supplemental\_FigureS1.tiff
- Supplemental\_FigureS4.tiff
- Supplemental\_FigureS2.tiff
- Supplemental\_FigureS5.tiff
- Supplemental\_FigureS3.tiff
- Supplemental\_FigureS6.tiff
