# Supplementary material for: Common microbehavioral “footprint” of two distinct classes of conditioned aversion
Source: Learn Mem. 2017 May;24(5):191–8. doi: 10.1101/lm.045062.117 (PMC5397685; doi:10.1101/lm.045062.117)
Supplement: Supplemental Material [file supp_24.5.191_Supplemental_Figure_Legends.docx]

**Supplementary Material**

**Supplementary movie**

The example track from Fig. 1 and its corresponding head vector angular speed, tail vector angular speed, heading angle, bearing and bending angle angle are shown as a movie.

**Figure S1: Behaviour under baseline conditions**

Larval behaviour in the absence of quinine, as well as in the presence of fructose. **(A)** Endpoint-counting Preference, **(B)** Run speed, **(C)** Run speed-modulation, **(D)** HC rate-modulation, **(E)** Reorientation per HC. Differences between reciprocals are not significant (*p >* 0.05, MWU). The notable exception is Reorientation per HC after aversive training (E, left boxes) (*p* < 0.05, MWU) which just hits significance. In summary, we conclude that larvae behave the same after paired and unpaired aversive training in the absence of quinine (with the exception in mind), as well as after paired and unpaired appetitive training in the presence of fructose. We therefore pooled the reciprocally trained groups in order to provide a ‘Baseline’ with no learned behaviour being expressed. Distinct ‘Baselines’ were used for aversive and appetitive training.

**Figure S2: Innate behaviour**

Given that we tested animals on different substrates, we asked whether the presence of either the sugar or the quinine substrate also had an influence on chemotaxis. The experiments and analyses follow the described procedures (see material and methods), yet we omitted the training.

**(A)** Olfactory preference was not affected by sugar or quinine substrate. On all three substrates larvae showed an equally small preference towards the odour (pooled data: *p* < 0.05, OSS).

**(B)** The presence of both fructose and quinine decreased run speed, similar to what was observed in trained animals (Fig. 3A, B).

**(C)** Run speed-modulation was not affected by sugar or quinine substrate, and not different from chance level (pooled data: *p* > 0.05, OSS).

**(D)** HC rate-modulation was significantly reduced in presence of sugar but not quinine.

**(E)** Reorientation per HC was significantly decreased in presence of quinine but not sugar.

In summary, the observed effects seem to be spurious and not consistent. We therefore hesitate to draw conclusions regarding the effects of sugar or quinine substrates. An exception is the reduction of run speed in presence of both sugar and quinine. This result fits well to the results after training both in the current (Fig. 3A-B) and our previous study (Schleyer et al. 2015b).

We find a significant difference across groups for run speed, HC rate-modulation and reorientation (*p* < 0.05, df = 2, N = 48, 28, 28, KW). In case of preference and run speed-modulation, no significant difference across groups was detected (*p* > 0.05, df = 2, N = 48, 28, 28, KW). Significant between-group differences (MWU, *p* < 0.05 corrected according to Bonferroni-Holm) are indicated with lower case letters above the boxes, significant differences to chance level (OSS, *p* < 0.05 corrected according to Bonferroni-Holm) are indicated by asterisks below the boxes.

**Figure S3: Sub-threshold HCs**

For our analysis, we take into account only HCs with an HC angle > 20°. In this figure, we show the respective analysis for HCs with an HC angle < 20° (in the following called sub-threshold HCs).

**(A)** Histogram of HC angles of all HCs in the data set. Open boxes display sub-threshold HCs that are analysed in this figure, filled boxes display HCs above threshold that are analysed in the rest of the study.

**(B)** HC rate-modulation after paired aversive training is lower than after unpaired aversive training. In absence of quinine, an intermediate HC-rate modulation is seen.

**(C)** HC rate-modulation after paired appetitive training is higher than after unpaired appetitive training. In presence of fructose, an intermediate HC-rate modulation is seen.

**(D)** Reorientation per HC is not affected by aversive training.

**(E)** Reorientation per HC is not affected by appetitive training.

In summary, the initiation of sub-threshold HCs follow the same rules as for their bigger counterparts (compare Fig. 4A-B), but the direction of sub-threshold HCs seem to be not modulated by conditioned aversion or approach. It is possible that modulations of HC direction are not well detected due to a bad signal-to-noise ratio. Therefore we restrict our analysis to bigger HCs that can be characterised with more confidence.

We find a significant difference across groups only for HC rate-modulation (*p <* 0.05, df = 2, N = 27, 56, 27, KW). In case of Reorientation per HC, no significant difference across groups was detected (*p >* 0.05, df = 2, N = 27, 56, 27, KW). Significant between-group differences (MWU, *p* < 0.05 corrected according to Bonferroni-Holm) are indicated with lower case letters above the boxes, significant differences to chance level (OSS, *p* < 0.05 corrected according to Bonferroni-Holm) are indicated by asterisks below the boxes.

**Figure S4: Basic properties of HCs**

Characterisation of (above threshold) HCs after aversive and appetitive training.

**(A, B)** HC rate after (A) aversive and (B) appetitive training measure how many HCs larvae perform per second. No differences between groups are observed. The HC rate underlies the calculation of the HC rate-modulation as displayed in Fig. 4A-B.

**(C, D)** Absolute HC angles after (C) aversive and (D) appetitive training measure the average size of HCs. After paired aversive training, HC angles are slightly bigger than under baseline conditions. No differences between groups are observed after appetitive training.

We find a significant difference across groups only for absolute HC angle after appetitive training (*p <* 0.05, df = 2, N = 27, 56, 27, KW). In all other cases, no significant difference across groups was detected (*p >* 0.05, df = 2, N = 27, 56, 27, KW). Significant between-group differences (MWU, *p* < 0.05 corrected according to Bonferroni-Holm) are indicated with lower case letters above the boxes.

**Figure S5: Run speed**

Colour-coded run speed as a function of bearing and distance to the odour source, separately for **(A)** aversive training and **(B)** appetitive training. Little effect appears to be exerted by either bearing direction or distance to the source on run speed. However, run speed does vary across experimental groups, in particular in relation to the presence or absence of sugar or quinine (Fig. 3A-B). For each condition, we pooled all data and calculated the run speed over all bearing angles ([-180°, 180°], where 0° represents a bearing towards the odour source), applying a sliding box filter of ± 60° and ± 23 mm at each step (step width of 6° and 2.3 mm).

**Figure S6: HC rate**

Colour-coded HC rate as a function of bearing and distance to the odour source, separately for **(A)** aversive training and **(B)** appetitive training. The HC rate varies with both bearing and distance to the odour source, as well as between experimental groups. In particular after unpaired appetitive training, larval behaviour changes strongly with distance to the odour (right-most panel in [B]): far from the odour, larvae perform more HCs while heading away from the odour source than while heading towards it. Near the odour source, however, they perform more HCs while heading towards the odour, indicating odour aversion. Therefore we restricted our analysis to a distance < 59 mm (the half-maximal distance to the odour source). For each condition, we pooled all data and calculated the HC rate over all bearing angles ([-180°, 180°], where 0° represents a bearing towards the odour source), applying a sliding box filter of ± 60° and ± 23 mm at each step (step width of 6° and 2.3 mm).

**Figure S7: HC angle**

Colour-coded HC angle as a function of bearing and distance to the odour source, separately for **(A)** aversive training and **(B)** appetitive training. The HC angle varies with both bearing and distance to the odour source, as well as between experimental groups. In particular after paired aversive training and unpaired appetitive training, larval behaviour changes strongly with distance to the odour (left-most panel in [A], right-most panel in [B]): far from the odour, larvae make more HCs towards the odour. Near the odour source, however, they make more HCs away from the odour, indicating odour aversion. Therefore we restricted our analysis to a distance < 59 mm (the half-maximal distance to the odour source). For each condition, we pooled all data and calculated the HC angle over all bearing angles ([-180°, 180°], where 0° represents a bearing towards the odour source), applying a sliding box filter of ± 60° and ± 23 mm at each step (step width of 6° and 2.3 mm).

**Figure S8: Time-resolved preferences**

Display of time-resolved preferences as seen in Fig. 2A’-B’. Here, we display medians (bold line) as well as the 25-75% quantiles (pale area).

**(A)** Aversive training. Top: paired training, middle: baseline, bottom: unpaired training.

**(B)** Appetitive training. Top: paired training, middle: baseline, bottom: unpaired training.
