# Supplementary figures and images for: Common microbehavioral “footprint” of two distinct classes of conditioned aversion
Source: Learn Mem. 2017 May;24(5):191–8. doi: 10.1101/lm.045062.117 (PMC5397685; doi:10.1101/lm.045062.117)

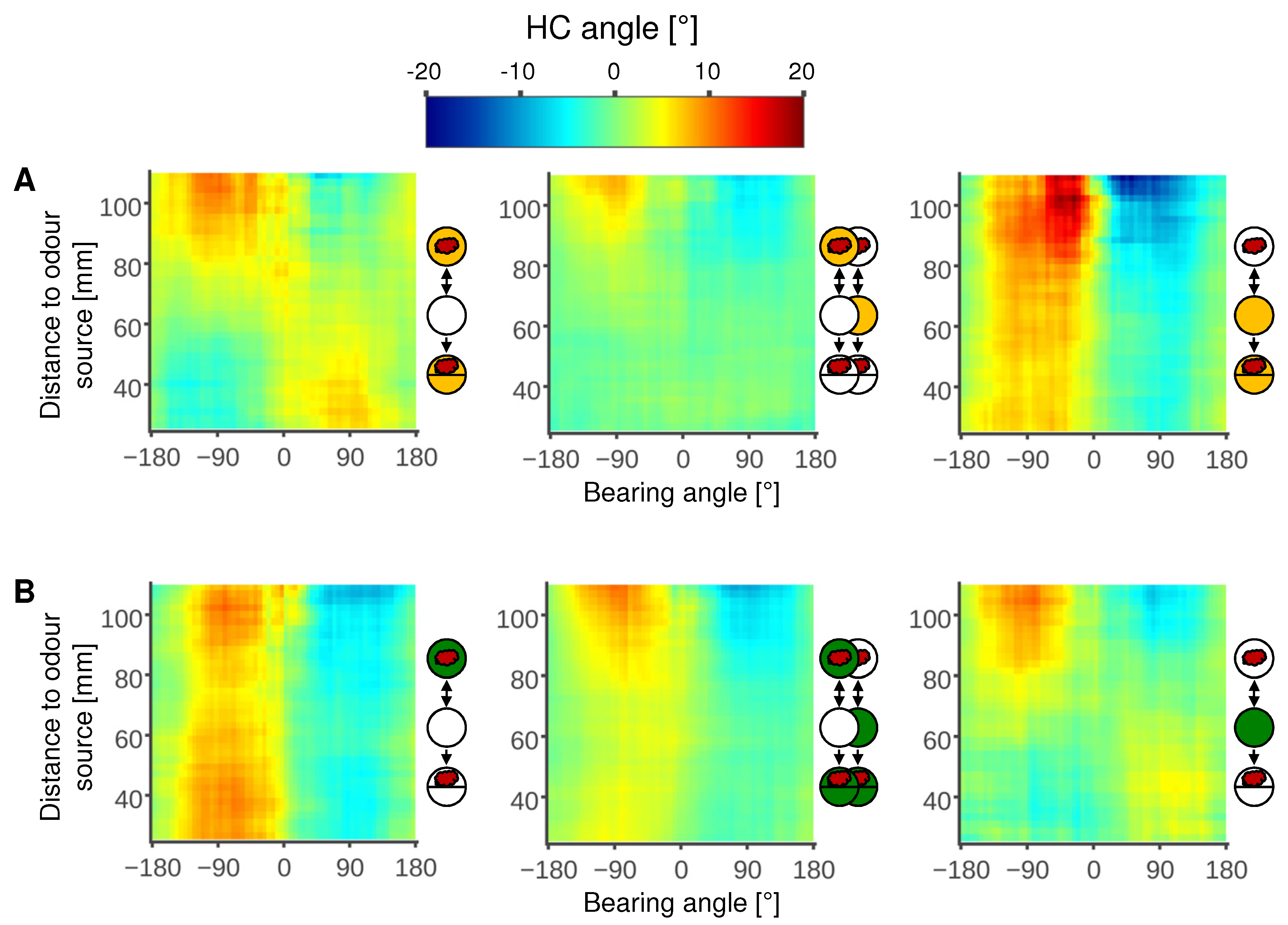

Supplement: Supplemental Material [file supp_24.5.191_Supplemental_FigureS7.tif]

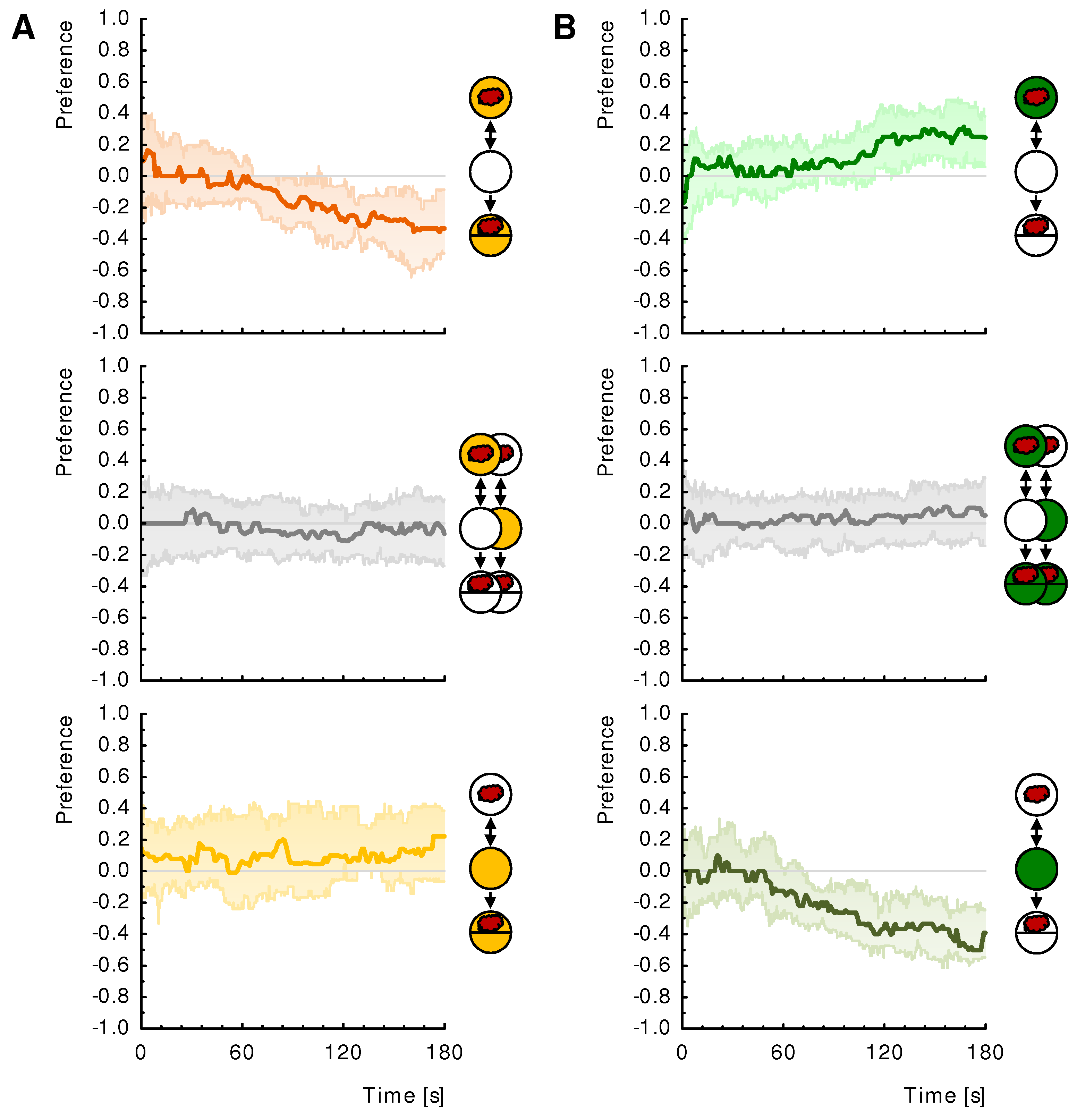

Supplement: Supplemental Material [file supp_24.5.191_Supplemental_FigureS8.tif]

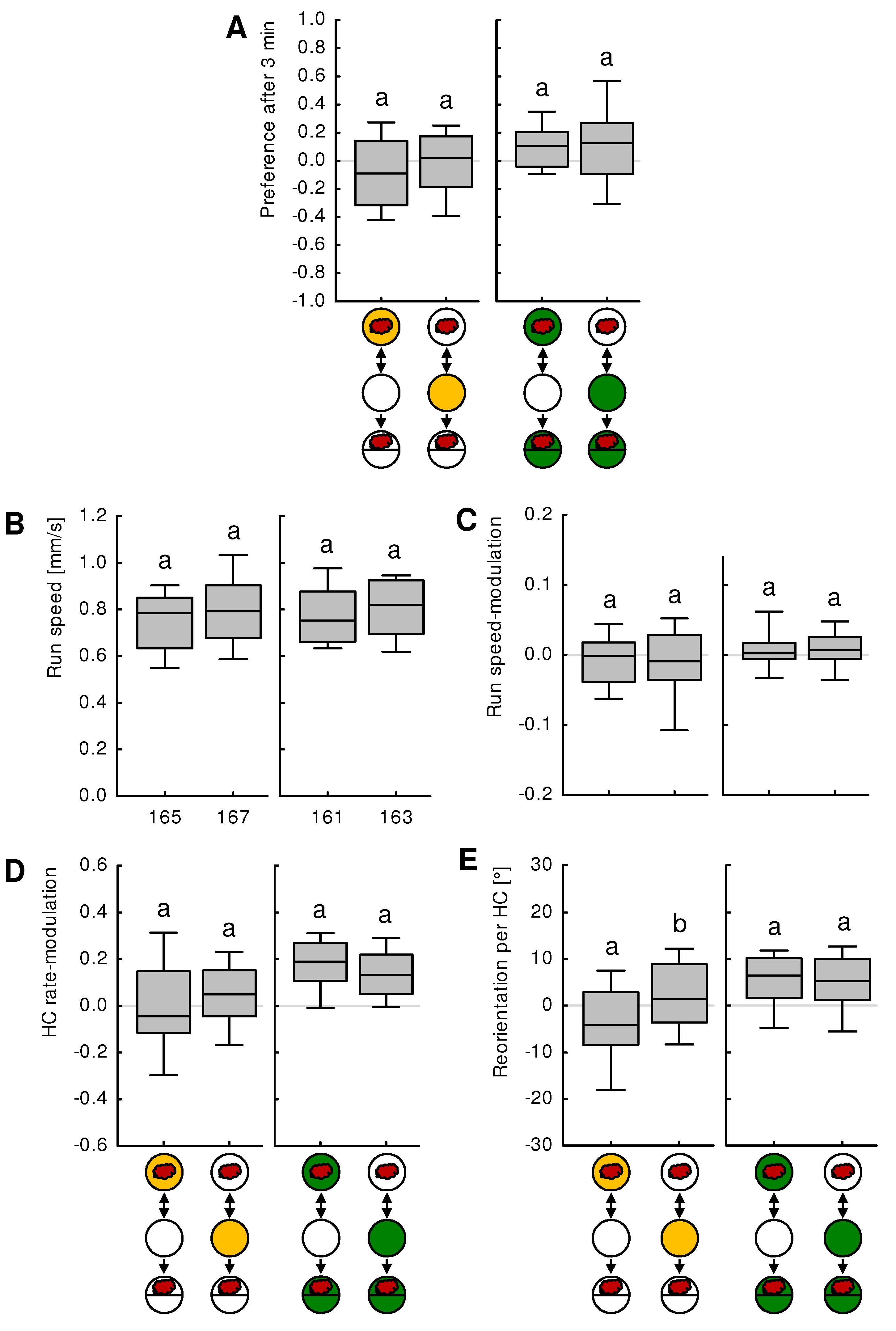

Supplement: Supplemental Material [file supp_24.5.191_Supplemental_FigureS1.tif]

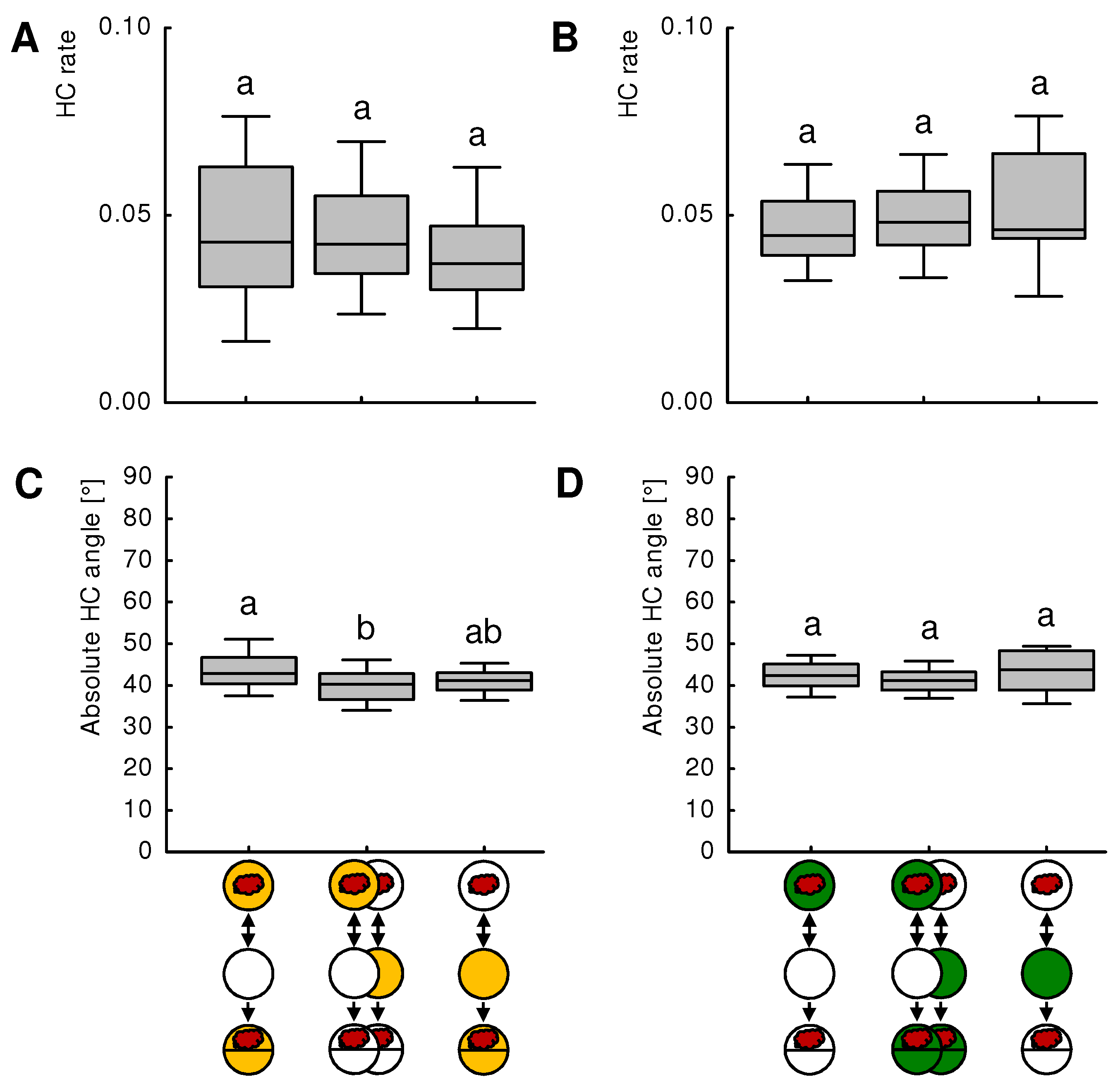

Supplement: Supplemental Material [file supp_24.5.191_Supplemental_FigureS4.tif]

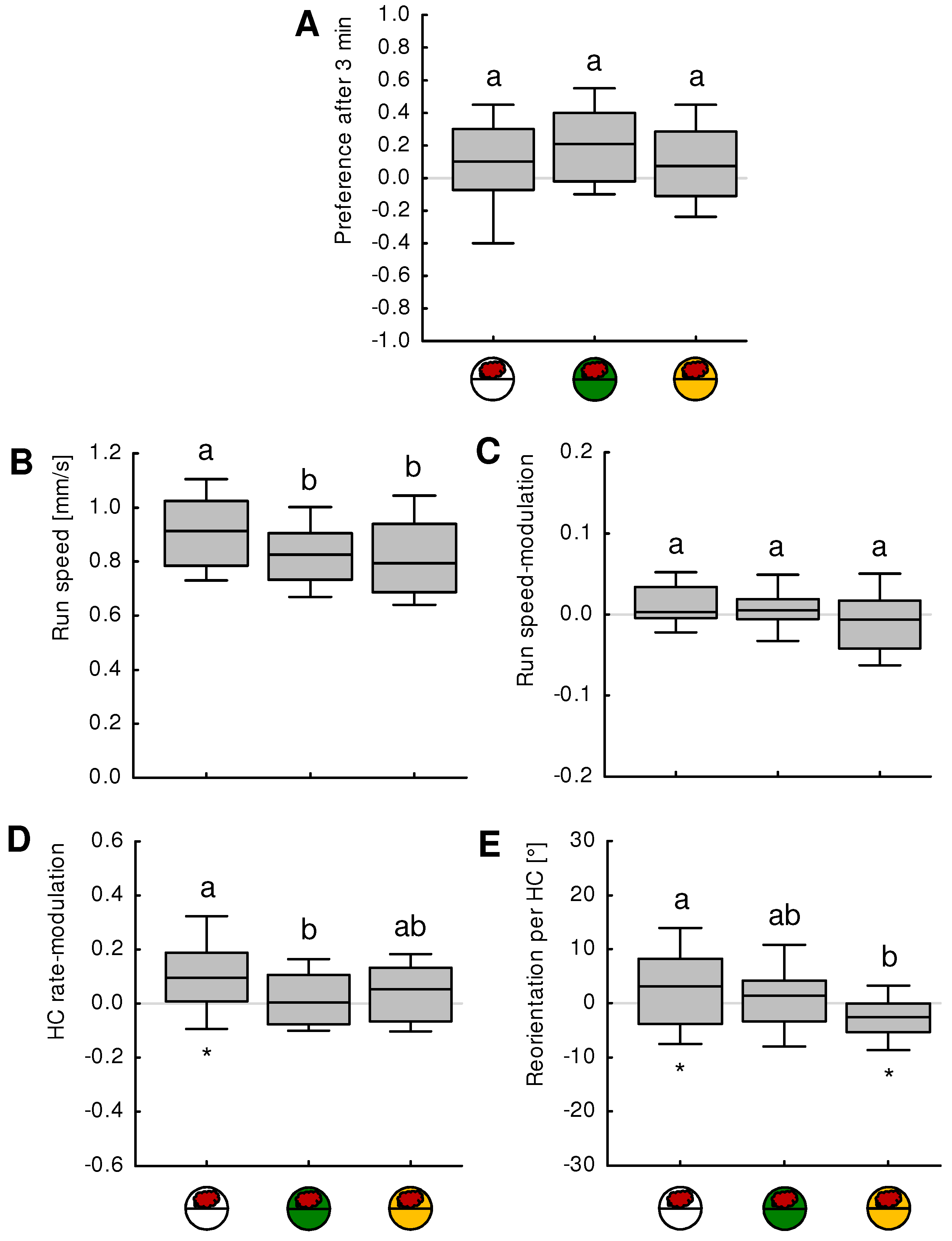

Supplement: Supplemental Material [file supp_24.5.191_Supplemental_FigureS2.tif]

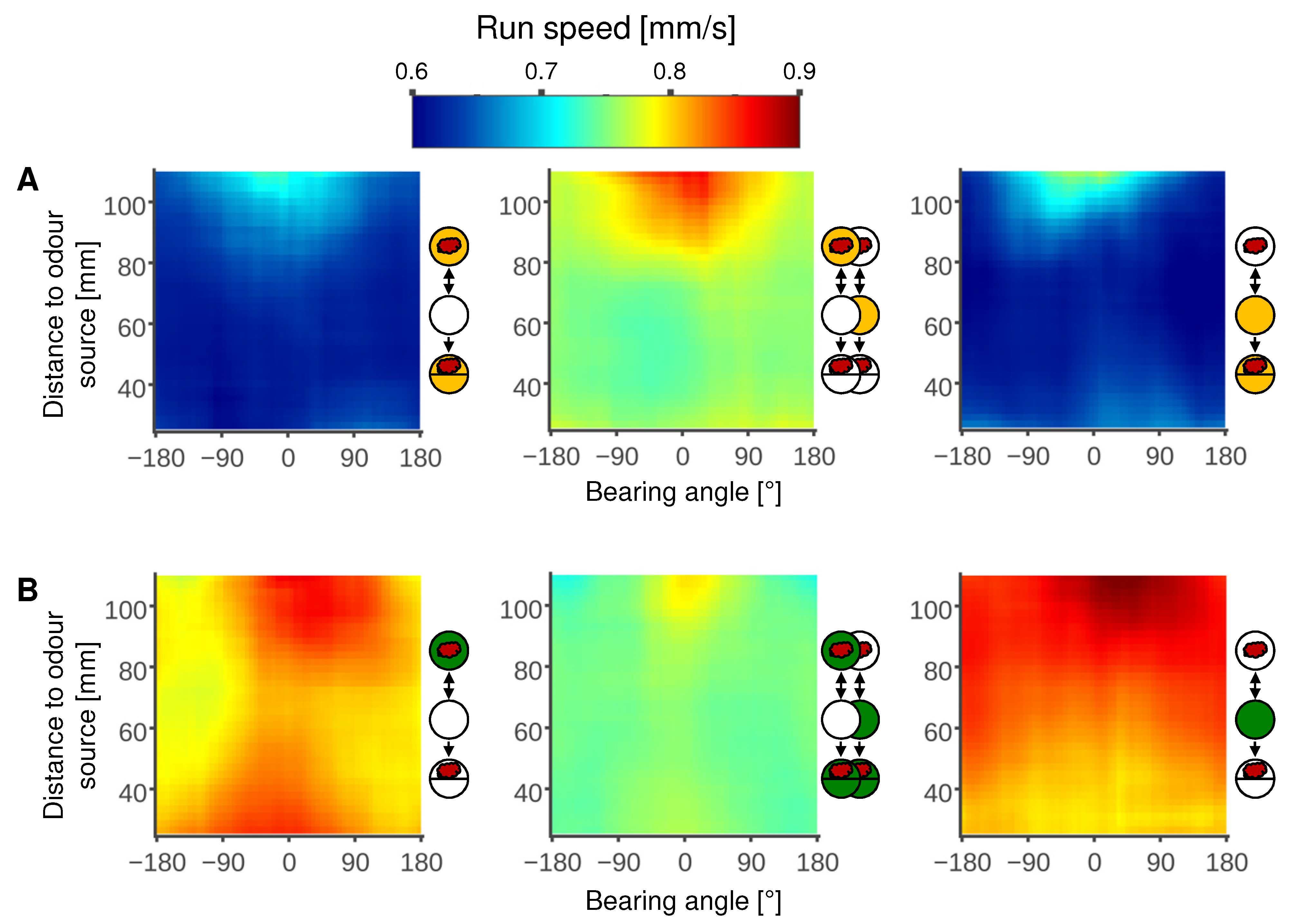

Supplement: Supplemental Material [file supp_24.5.191_Supplemental_FigureS5.tif]

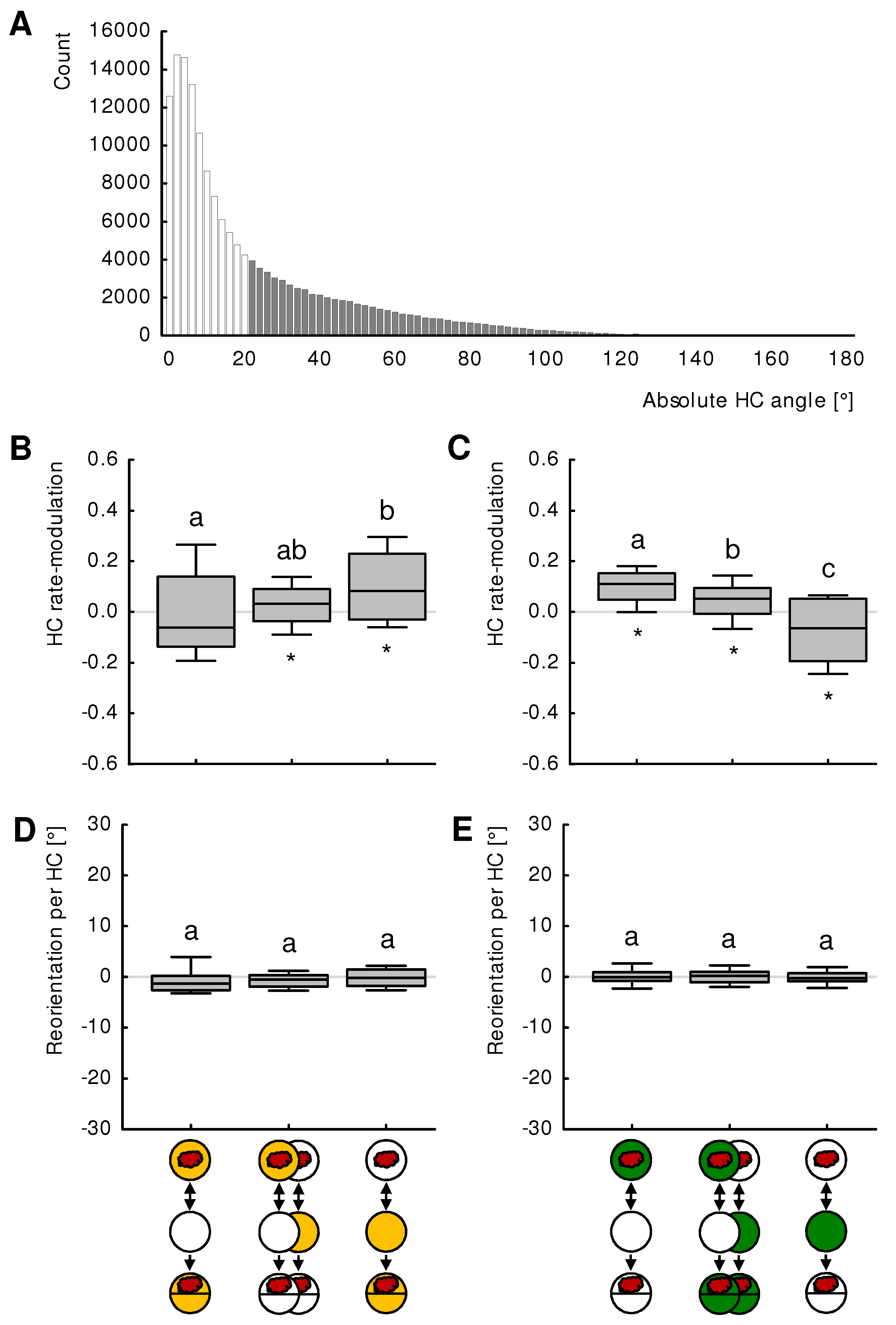

Supplement: Supplemental Material [file supp_24.5.191_Supplemental_FigureS3.tif]

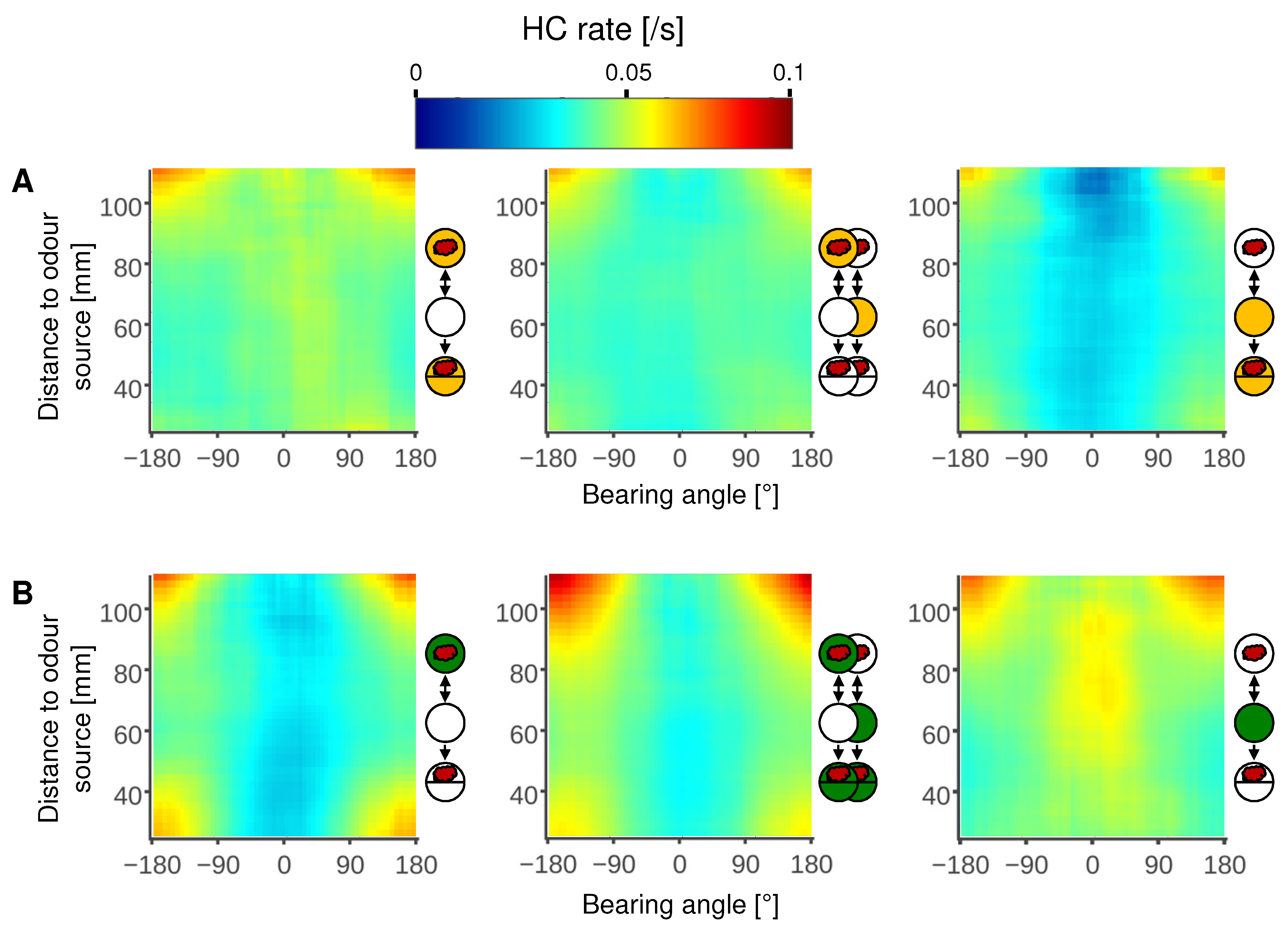

Supplement: Supplemental Material [file supp_24.5.191_Supplemental_FigureS6.tif]
